# Practical Points

**Published:** 1893-06

**Authors:** Henry Barnes


ARTICLE IV.

### PRACTICAL POINTS.

BY DR. HENRY BARNES.

Pink gutta-percha is to be found in every well appointed dental office, and is probably used for more purposes than any other one material in our possession. A few are here mentioned: As temporary fillings in cavities already prepared, when want of time or other causes prevents the insertion of a permanent filling; as temporary fillings in large cavities not prepared, to prevent nerve exposure till such time as patient or operator may find time to devote to permanent filling; as a wedge between teeth separated by cotton, rubber or other means to allow inflammation to subside and retain space during the operation of permanent fillings; as a wedge between teeth, having large spaces from extraction or otherwise, standing next to the one being filled, to distribute the force of the mallet blow, and thus securing to the patient the greatest possible comfort; as a rest under the arm of the Perry separator to secure the instrument from rocking, slipping, etc.; as a separator and rest for the jaws during operations; as cones to force chloro-percha into every portion of the nerve chamber. It may also be used to make small handles for the broach, thus securing a better hold and more perfect manipulation of the broach during nerve extraction, especially when the approach is from the distal surface; also wound around the ends of a ribbon saw and making a secure handle for this instrument; also for separating teeth held on the end of an instrument, and heated to determine the life or death of the dental pulp.

Used as chloro-percha (gutta percha dissolved in chloroform) to fill root canals and making the most perfect filling for this purpose (universally used) which has yet been brought to our notice; to repair the break in the rubberdam; to secure remedies, within cavities, when mixed with cotton. We injure our hand or fingers, and at such times, if we immediately suck the wound and wash with bichloride mercury solution 1-1000, thoroughly dry and smear over with chloro-percha, all danger from infection is removed, and the wound will immediately heal. For filling some of the molars when it is impossible to apply the dam, a band of German silver or other metal secured on the inside with chloro-percha, and then forced over the tooth, will prevent the moisture from interfering with the work; it is also an excellent article to flow over the inside of a cavity when the nerve is nearly exposed and when it is desired to cap with cement.

AMALGAM.—To repair a break in a tooth next a gold filling on the lingual surface, also to repair when decay has attacked the tooth at the cervix beneath the filling. In the first place take a little scrap of amalgam already mixed, and which has crystallized, then heat a spatula quite hot and press the amalgam into place; as it quickly becomes hard, no danger is feared for immediate dislodgement. This is especially useful where it would loosen the gold filling if much cutting were done. After thoroughly preparing the cavity, fill as usual. Generally decay will be retarded for years. This is one of the condemned practices of twenty years ago. So our idols are broken.

Fillings at the lingual cervix, and in the lingual pit and groove of the lateral incisors: Take a piece of tin or other metal ½ inch wide and long enough to surround the tooth to be filled, fasten the end with thread or other means, pass over the tooth, allowing the metal to slide over the cavity between the gum and tooth, and with a burnisher crowd down and bend over the edge as much as possible. This will expose the cavity to view, and crowd the gum from the tooth, thus allowing of freedom during the operation, and it will also prevent moisture getting into the cavity. A thin coating of chloro-percha applied with a nerve broach will greatly assist in some cases. This method is useful in filling cavities on the distal surface of third molars where the gum has grown over the distal ridge.

EXTRACTING NERVES.—It frequently happens that the nerves in the lingual root of the upper molars and distal root of lower molars are difficult to extract. I have been using two or three broaches in a bunch for such cases with good results. Have we a prescribed procedure for filling nerve canals?

After arsenic has been in the tooth for three days, I remove the capping and drill into the pulp chamber, and after removing the pulp and nerves, or as much of them as I can, apply oil of cassia on a pledget of cotton and dismiss my patient for a day, then after dressing with peroxid-hydrogen, I use oil of cassia and dry out with very hot air and immediately fill.

THE USE OF THE SEPARATOR IN FILLING TEETH.—Perhaps the best of any yet introduced is the Perry. It saves the time of all concerned, and if judiciously used is a boon to our patients. With it there are few cases which can not be separated enough for immediate filling. After the cavities are filled a little more space may be gained, so that natural contour is preserved, and food is not crowded into the V-shaped spaces to the great inconvenience of the patient, and very likely to re-decay at an early day. This is a nice point.

Sand paper discs, if coated with soap, instead of sweet oil or glycerin, will not heat the tooth so quickly as when nothing is used, and gives a more polished surface to the filling.

THE APPLICATION OF THE RUBBER-DAM.—I have never been able to understand how any thing is gained by using small pieces of rubber-dam. It should be large enough to well cover the mouth, cheeks and chin, so that it may be held and kept out of the way during operations. Many breaks about the necks of the teeth after the dam has been applied is due to punching the holes too near together. They should be punched far enough apart so that the rubber will not be stretched in the interdental spaces, and be sure to punch enough holes. The ligating is easily done by allowing the thread to extend from tooth to tooth on the lingual side without a single knot. Black carpet thread makes a good ligature, and its color makes it possible to detect any fibers which may have caught on the cervical border of a cavity, especially in deep cavities, splitting the thread and using two strands for teeth closely wedged.—*Ohio Dental Journal.*

